# Review of knee arthroscopy performed under local anesthesia

**DOI:** 10.1186/1758-2555-1-3

**Published:** 2009-01-19

**Authors:** Billy Kan-Yip Law, Patrick Shu-Hang Yung, Eric Po-Yan Ho, Joseph Jeremy Hsi-Tse Chang, Grace Ken Kwok, Kwai-Yau Fung, Kai-Ming Chan

**Affiliations:** 1 Department of Orthopaedics and Traumatology, Prince of Wales Hospital, Faculty of Medicine, The Chinese University of Hong Kong, Hong Kong, PR China; 2 The Hong Kong Jockey Club Sports Medicine and Health Sciences Centre, Faculty of Medicine, The Chinese University of Hong Kong, PR China; 3 Department of Orthopaedics and Traumatology, Alice Ho Miu Ling Nethersole Hospital, Hong Kong, PR China

## Abstract

Local anesthesia for knee arthroscopy is a well documented procedure with diagnostic and therapeutic role. Numerous therapeutic procedures including partial menisectomy, meniscus repair, abrasion chondroplasy, synovectomy, loose body removal can be performed safely and comfortably. Appropriate case selection, anesthetic strategy and technical expertise are the key to smooth and successful surgery.

## Review

### Introduction

Local anesthesia (LA) for knee arthroscopy is a well documented procedure [1,2] that offers several potential advantages over other types of anesthesia:

1. Cost is low and cost effective

• Randomized control study by Forssblad M [3] showed that the total hospital time, recovery time were significantly shorter for LA arthroscopy when compared with spinal anesthesia or general anesthesia. The total cost was also significantly lower.

2. Patient is awake and can follow the procedures.

3. Complications are rare

• Potential complications of general anesthesia are eliminated e.g. aspiration, malignant hyperthermia

However, in some cases, conversion to general anesthesia (GA) is necessary, mainly because of patient pain tolerance that makes complete examination impossible or the procedure is too complicated to be done under local anesthesia. [4]

In order to perform successful arthroscopy under local anesthesia, case selection, anesthetic choice and technical details are important factors to be considered.

### Case selection

Numerous procedures can be performed successfully under local anesthesia [5]. These include:

1. Partial menisectomy (both medial and lateral meniscus)

2. Meniscus repair with all inside technique (e.g. FasT-Fix (Smith Nephew Endoscopy, Andover, MA, USA))

3. Removal of loose body

4. Debridement

• Synovecotmy

5. Plica excision

6. Cartilage procedures

• Abrasion chondroplasty

• Microfracture of femoral condyle

And concerning the pain experienced during arthroscopy, Dye [6] has reported on the neurosensory mapping of the internal structure of the knee without anesthesia. They demonstrated that severe pain was reported during probing of the suprapatellar capsule, meniscal capsular margin, infrapatellar fat pad, and the insertion site of the cruciate ligament. Minimal pain was reported while probing cartilage and inner rim of the meniscus.

And according to Takahashi T [7], local anesthesia provided good pain control during partial menisectomy, chondroplasty and removal of loose body. Patients sometimes experienced more pain during treatment of the suprapatellar pouch, including the plica and the anterior cruciate ligament.

Hypertrophic synovitis (presented as capsular swelling and diagnosed on clinical examination) is a relative contraindication since administration of LA is quite painful for patients with extensive synovitis and the surface of the synovium becomes larger when it is inflamed and the standardized dosage may not have been sufficient to produce adequate anesthesia [2].

Gross deformity, e.g. severe varus or valgus knee with narrowing of joint space also makes complete examination difficult.

Absolute contraindication of local anesthesia arthroscopy include allergy to local anesthesia or local infection at selected portal and injection sites.

### Anesthetic choice

The most commonly used regime is a combination of intraarticular and portal site injection of local anesthetic with adrenaline. Intravenous sedation may be used in apprehensive patients.

The safety dosage has been established by Weiker GG etal. [8]. Fifteen healthy patients were included. 25 ml of 1% lidocaine with epinephrine (1:100,000) and 25 ml of 0.25% bupivacaine were instilled into the knee joint. An additional 40 ml of the combined solution was used to anesthetize four arthroscopic portal sites from the skin into the joint capsule. Arthroscopy was then performed. Blood samples from 5, 15, 30, 60, and 120 minutes after intraarticular injections were taken. Levels of the anesthetic agents in all patients at all time intervals were well within the safety range. And no complications from the anesthetic agents were noted in over 500 similar cases.

Some authors tried to use a minimal dosage of anesthetics. Iossifidis [9] has performed 53 knee arthroscopies under low volume (20 ml) local anaesthesia using half the recommended safe dose of anesthetic agents (10 ml 0.5% bupivacaine + 10 ml 2% lignocaine with adrenaline 1:200,000), as a single intra-articular injection (10 ml) together with skin infiltration of the arthroscopic portals (5 ml to each portal). 62 lesions were diagnosed and 48 surgical procedures were successfully carried out in 53 patients. 97% of patients were satisfied with the procedure which caused little or no discomfort in 94% of cases. The combination of short acting and long acting anesthetic agent offers advantage of controlled anesthesia localized to the knee joint and provides prolonged post operative analgesia.

For apprehensive patients, intravenous sedatives (e.g. Midazolam) can be given. But this requires intraoperative monitoring of oxygen saturation and vital signs. Resuscitation equipment and antidote (Flumazenil) should be available.

Miskulin M [10] had used diclofenac (1 mg/kg) as preemptive agent. The theoretical advantage is that preemptive administration of diclofenac can reduce hyperalgesia by inhibiting cyclooxygenase (COX) and decreasing tissue prostaglandin synthesis and may reduce analgesic requirement. In a series of 628 patients, 10 ml 2% lidocaine with 1:200,000 epinephrine was injected into the joint cavity, and 5 ml of 2% lidocaine with 1:200,000 epinephrine was injected into each portal site, all with preemptive diclofenac injected intravenously just before the procedure. Arthroscopy was well tolerated by 98.5% of patients and only 1.4% of procedures had to be terminated prematurely because of patient discomfort.

In general, most patients tolerate the procedure well without any complication. However, vital signs should still be monitor during the procedure and an intravenous access should be available before the procedure as a minority of patient may develop vasovagal attack.

Last but not the least, detail explanation of procedures before the operation and communication during the procedure will alleviate most of the anxiety.

### Technical tips

#### Injection of local anesthetics

During injection of local anesthetics to the portal sites, majority of the drug should be injected into the subcutaneous layer instead of the subcapsular layer, otherwise the fat pad will be pushed into the joint and making initial visualization difficult.

During intraarticular injection of local anesthetics, do make sure that it was injected into the joint and not into the subcutaneous tissue, especially for muscular patients. Bulging from the medial gutter and a positive patellar tap sign will be noted after successful intraarticular injection.

Do wait for ~20 minutes after injection before the procedure starts.

#### Trocar cannula

Do use a cannula with both inflow and outflow, such that an extra supralateral outflow portal is not necessary. Since according to study by Takahashi T [7], pain experienced at the time of local anesthetics injection was more severe than pain experienced during the surgical procedure.

During introduction of trocar cannula, initial insertion into the patello-femoral joint is not a must since this will also cause pain, especially when the patient is not relaxed and the quadriceps is contracted. It may be inserted into the femoral notch first and then fluid is instillated and then the PFJ can be entered more easily.

#### Haemostasis

For most of the procedures, tourniquet is not necessary for experienced surgeons. But it may be placed as a standby manner, such that it may be inflated for short period of time if there is really difficult bleeding that make visualization difficult.

Adrenaline may be mixed into the irrigation fluid (10 ml of 1:10,000 adrenaline into 3 L of irrigation fluid), usually the first bag, can help in haemostasis.

If difficult bleeding is encountered, the pressure from the water column from the arthroscope is usually sufficient to achieve temporary haemostasis, and identification of bleeding source. Then radiofrequency can be used for haemostasis.

### Pain during procedure

#### Synovectomy

Patient will also experience pain during synovectomy, so extensive synovitis is a relative contraindication of LA arthroscopy.

Most patients tolerate well for partial menisectomy, shaving of plica, loose body removal or abrasion chondraplasty.

#### Experience from a teaching hospital

From July 2003 to June 2005, 190 patients underwent day case knee arthroscopy in Prince of Wales hospital. All procedures were performed under local anaesthesia: using 30 ml (20 ml intra-articular and 5 ml to each portal site) of 1% lignocaine in 1:200,000 adrenaline. There was no complication related to the injection of local anesthesia. 35% were diagnostic procedures and 65% were therapeutic. Therapeutic procedures included partial menisectomy, all inside meniscal repair, removal of loose bodies, microfracture, abrasion chondroplasty, debridement in knee osteoarthritis and shaving of plica. The average operating time was 29.2 minutes (range 12 – 75 minutes). Only 2 cases required a tourniquet during the procedure. Sedatives were not required in all cases. The average intraoperative VAS as reported by the patient was 3.1 (range 1–9). Discomfort was mainly felt during initial introduction of the trocar cannula, and upon shaving of severely inflamed synovium. 3 cases had to be abandoned during the procedure due to patient intolerance from anxiety & pain, as well as tight joint space from advance osteoarthritis. 95% of the patients agreed to have the same procedure performed under local anesthesia in the future.

## Conclusion

Knee arthroscopy under local anesthesia is a safe, well tolerated and cost effective alternative to conventional techniques.

## Competing interests

The authors declare that they have no competing interests.

## Authors' contributions

BKYL reviewed the literature and drafted the manuscript. KMC, PSHY, EPYH, BKYL, JJHTC, GKK did the surgeries and did the statistics analysis. KMC, KYF participated in coordination.
